# Can Artificial Sweeteners Increase the Risk of Cancer Incidence and Mortality: Evidence from Prospective Studies

**DOI:** 10.3390/nu14183742

**Published:** 2022-09-10

**Authors:** Shoumeng Yan, Feifei Yan, Liping Liu, Bo Li, Shuxiang Liu, Weiwei Cui

**Affiliations:** 1 School of Nursing, Jilin University, Changchun 130021, China; 2 Department of Nutrition and Food Hygiene, School of Public Health, Jilin University, Changchun 130021, China; 3 Department of Immunology, College of Basic Medical Sciences, Jilin University, Changchun 130021, China; 4 Department of Epidemiology and Biostatistics, School of Public Health, Jilin University, Changchun 130021, China

**Keywords:** artificial sweeteners, cancer, incidence, mortality, meta-analysis

## Abstract

Cancer has become a major challenge in the global disease burden. Artificial sweeteners are a class of chemical compounds that are used as food and beverage addition agent to replace sugar. However, the health effects of consuming artificial sweeteners are still unclear. This meta-analysis was performed to evaluate the role of artificial sweeteners on cancer. The databases PubMed, Cochrane Library, MEDLINE, Web of Science and EMBASE were searched up until July 2022. A Newcastle–Ottawa scale (NOS) was used to estimate the study quality. A total of 25 observational studies were included with a total of 3,739,775 subjects. The intake of artificial sweeteners had no apparent association with overall cancer incidence and mortality. However, in Europe, artificial sweeteners’ intake could increase the risk of cancer incidence (HR/RR = 1.07, 95% CI = [1.02, 1.12], *I*^2^ = 25.8%, P = 0.223), which appears to be related to a shift in nutritional behaviors in the countries. Significant results were also observed in subgroups with aspartame and a mixed intake of artificial sweeteners. Moreover, higher risk was observed for artificial sweeteners intake in all-cause mortality (HR/RR =1.13, 95% CI = [1.03, 1.25], *I*^2^ = 79.7%, *p* < 0.001) and a J-shaped association between them was found. More data from well-conducted studies and clinical trials are required.

## 1. Introduction

Cancer has become a major challenge in the global disease burden. A global estimate of 23.6 million new cancer cases and 10.0 million cancer deaths occurred in 2019, which represented a 26.3% and 20.9% increase in new cases and deaths, respectively [1]. It is estimated that the burden of cancer will continue to increase over the next two decades at least [1]. The existing evidence suggests that obesity and cardiovascular disease (CVD) are directly or indirectly promoted by a high-sugar diet [2]. The similar effects of a high-sugar diet are also observed in the rates of cancer [3,4,5,6]. Therefore, as a substitute for sugar in foods and beverages, sweeteners have become more prevalent over the past few decades [7]. High-intensity sweeteners approved by the Food and Drug Administration (FDA) include six artificial sweeteners (saccharin, aspartame, acesulfame potassium (Ace-K), sucralose, neotame and aspartame) and two natural sweeteners (stevia (steviol glycosides) and Monk fruit) [8]. Since natural sweeteners are still in the developing stage in terms of sensory attributes, dominance duration and extraction technology, artificial sweeteners are still widely used [9]. Some of the metabolic and hormonal changes caused by high-calorie sugars either do not occur or are reduced after consuming artificial sweeteners [10]. Studies have indicated that the release of various hormones and markers of postprandial glucose homeostasis, such as insulin and glucagon-like peptide-1 (GLP-1), were not significantly altered when artificial sweeteners are delivered directly to the stomach or intestine. Artificial sweeteners alone do not stimulate insulin or incretin release [11,12,13]. In fact, the health and metabolic effects of consuming artificial sweeteners are unclear, and the debate about whether artificial sweeteners themselves increase cancer risk is still not solved. As early as 1970, based on the results of animal experiments, the FDA have suspected that cyclamate (sodium cyclohexyl sulfamate) could induce cancer, thus banning its use in all dietary foods and fruits in the United States [7].

Nowadays, according to the latest report, there is at least a third of adults and children who consume artificial sweeteners regularly in the United States, Europe and Australia [14]. It is due to the increasing availability of new artificial sweeteners and the rising obesity epidemic, thus more “low-calorie” products, especially beverages, are being used [15,16]. Therefore, artificial sweeteners have again been linked to cancer. Although the effect of consumption of artificial sweeteners on gastrointestinal cancers and the intake of soft drinks on the mortality of cancer have been examined via meta-analysis, it is unclear whether artificial sweeteners cause cancer in any form [17,18]. Our study aimed to evaluate the role of artificial sweeteners on the risk of cancer incidence and mortality and all-cause mortality based on the data from all of the current prospective cohort studies.

## 2. Materials and Methods

### 2.1. Sources and Methods of Data Retrieval

The PubMed, Web of Science, MEDLINE, Cochrane Library and EMBASE databases were searched up to July 2022, and the used keywords included artificial sweetener, aspartame, artificially sweetened beverage (ASBs), cancer and tumor, to screen and identify published literatures. The search had no restriction on publication date or language. The detailed search strategy of PubMed that was performed is shown in Table S1.

### 2.2. Inclusion Criteria

**Figure 1:**
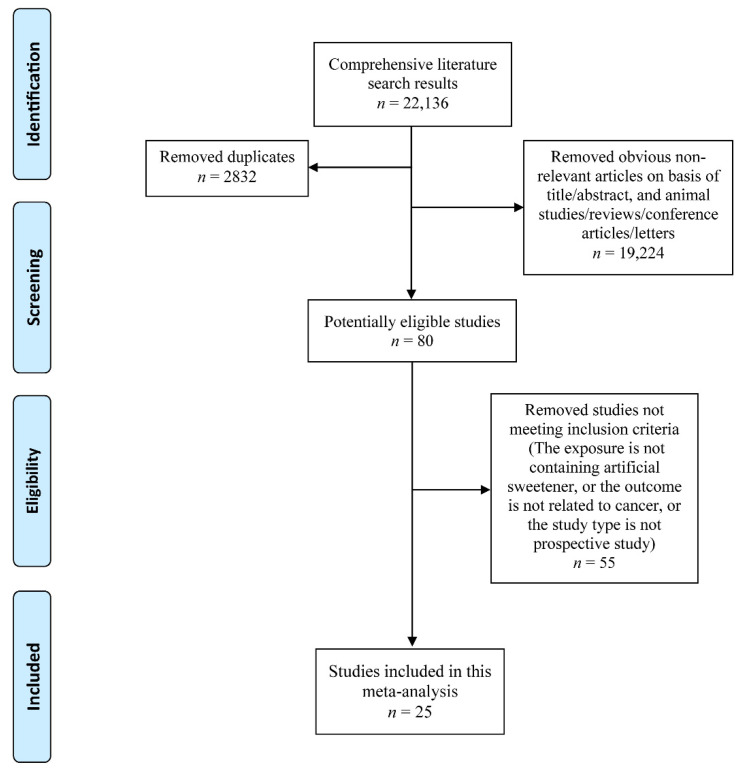
**Flow diagram of the literature search and selection**.

The inclusion criteria were as follows: (1) exposure: using artificial sweeteners in food, drinks and packets; (2) overall or site-specific cancer incidence or mortality, or all-cause mortality as a consequence; (3) hazard ratio (HR) or risk ratio (RR) with 95% confidence interval (CI) for the association between artificial sweetener and any type of cancer incidence and mortality was estimated; (4) animal studies, in vitro studies, duplicates, reviews, or conference papers were excluded. Two researchers independently assessed all of the included studies, resolved disagreements via discussion and collected final eligible literatures (Figure 1).

### 2.3. Data Extraction and Risk of Bias within Individual Studies

A literature review was conducted to extract the following data: publication year, first author, region, cancer type, usage of artificial sweeteners (including type and dose) and outcome of studies. Based on the Newcastle–Ottawa scale (NOS), the bias risk for the included study was estimated [19].

### 2.4. Statistical Analysis

Statistical analysis was carried out with the software Stata12.0. Statistics heterogeneity was evaluated using the *I*^2^ statistic and *p* < 0.05 was considered significant. In our study, random effects models were used in all of the analyses based on the level of heterogeneity. Egger’s test was employed to gauge publication bias, while the trim-and-fill approach was used to correct results and analyze the influence of bias on them [20,21]. Subgroup analyses were performed based on the region (Europe, Americas and Oceania), cancer type (obesity-related cancers and non-obesity-related cancers), type of artificial sweetener intake (ASBs alone or mixed intake (including drinks, packet and other methods)) and role of aspartame intake alone. Specially, all of the malignancies that include obesity as one of the risk factors or protective variables in their genesis are considered to be obesity-related cancers including colorectal cancer, gastric cancer, breast cancer, liver cancer, etc. Moreover, meta-regression was also conducted to confirm the source of heterogeneity.

## 3. Results

A total of 25 studies met our inclusion criteria, which contained 3,739,775 subjects (Table 1) [22,23,24,25,26,27,28,29,30,31,32,33,34,35,36,37,38,39,40,41,42,43,44,45,46]. Among them, the relationship between artificial sweeteners and cancer incidence was analyzed in 14 studies, and the risk of artificial sweeteners intake for cancer or all-cause mortality was assessed in the rest of the papers. The average score of NOS in all of the included studies was 8.52 (range 7–9).

### 3.1. Risk of Cancer Incidence

**Figure 2:**
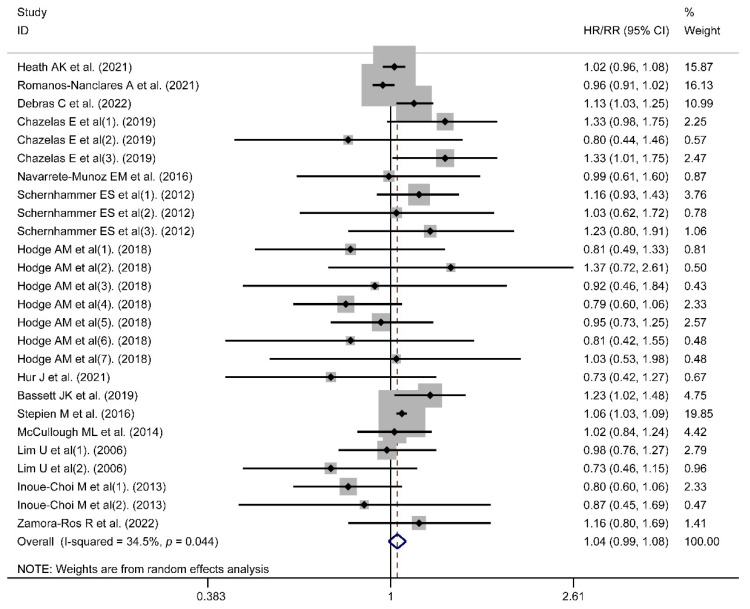
Meta-analysis results of artificial sweeteners intake for cancer incidence (HR: hazard ratio; RR: relative risk; CI: Confidence Interval) [22,23,24,25,26,27,28,29,30,31,32,33,34,42].

**Figure 3:**
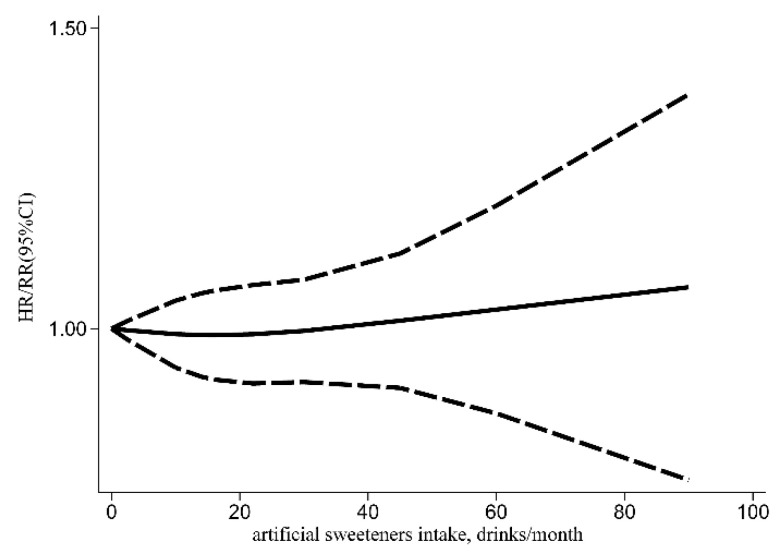
Dose–response relationship of artificial sweeteners intake with cancer incidence (HR: hazard ratio; RR: relative risk; CI: Confidence Interval).

A meta-analysis was performed in 14 included studies to evaluate the link between artificial sweeteners intake and cancer incidence. The results indicated that no association existed (HR/RR = 1.04, 95% CI = [0.99, 1.08], *I*^2^ = 34.5%, *p* = 0.044; Figure 2). Simultaneously, publication bias was not found in the results of cancer incidence (Egger’s test: coefficient = −0.234, *t* = −0.77, *p* = 0.450). According to the subgroup analysis of region, artificial sweeteners’ intake could increase the risk of cancer incidence in the European group (HR/RR = 1.07, 95% CI = [1.02, 1.12], *I*^2^ = 25.8%, *p* = 0.223). Meanwhile, artificial sweeteners’ mixed intake could increase the risk of cancer incidence (HR/RR = 1.09, 95% CI = [1.01, 1.18], *I*^2^ = 0.0%, *p* = 0.513). Moreover, significant differences were also observed with aspartame (HR/RR = 1.10, 95% CI = [1.01, 1.19], *I*^2^ = 0.0%, *p* = 0.467) (Table 2). The outcomes of the meta-regression showed that the regional factor could influence the total heterogeneity and effect size (*p* < 0.05) (Table 3). As a result, the regional factor may be the primary source of heterogeneity, as indicated by the results of the meta-regression and subgroup analyses. The dose–response relationship between artificial sweeteners intake and cancer incidence is shown in Figure 3.

### 3.2. Risk of All-Cause or Cancer Mortality

**Figure 4:**
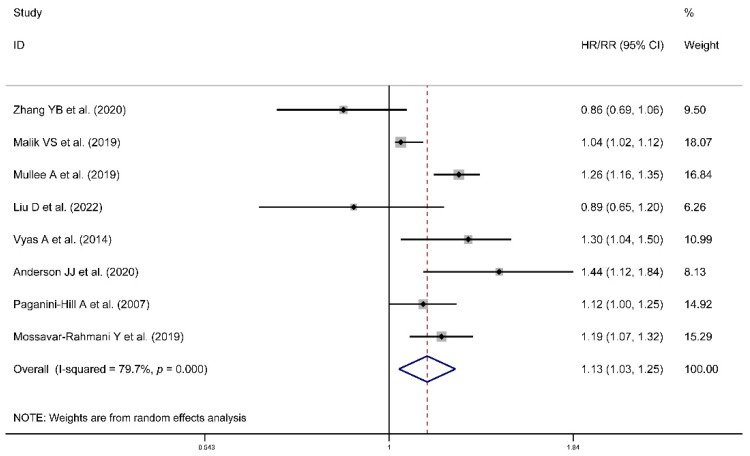
Meta-analysis results of artificial sweeteners intake for all-cause mortality (HR: hazard ratio; RR: relative risk; CI: Confidence Interval) [37,38,39,41,43,44,45,46].

**Figure 5:**
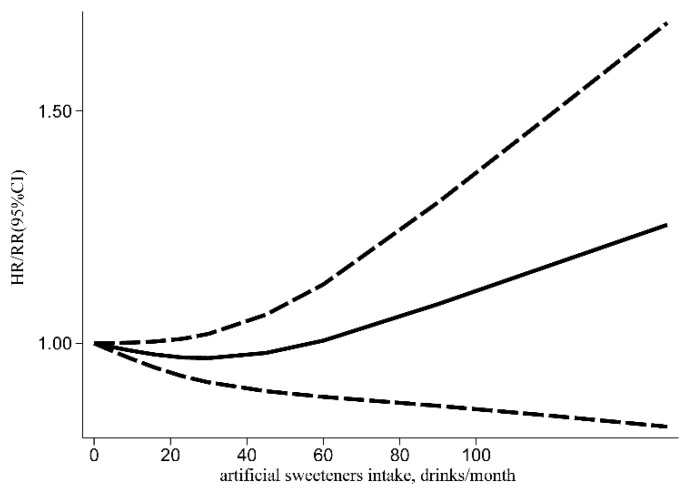
Dose–response relationship of artificial sweeteners intake with all-cause mortality (HR: hazard ratio; RR: relative risk; CI: Confidence Interval).

**Figure 6:**
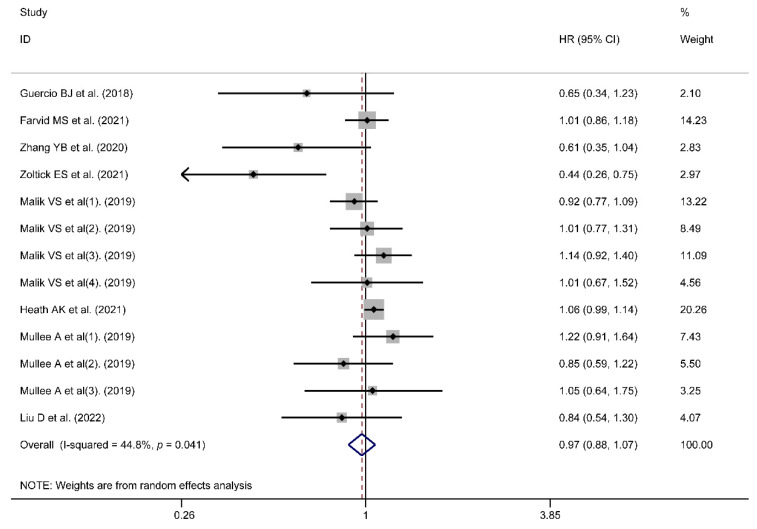
Meta-analysis results of artificial sweeteners intake for cancer mortality (HR: hazard ratio; CI: Confidence Interval) [26,35,36,37,38,39,40,41].

Higher risk was observed for artificial sweeteners’ intake in all-cause mortality (HR/RR = 1.13, 95% CI = [1.03, 1.25], *I*^2^ = 79.7%, *p* < 0.001; Figure 4) and a J-shaped association between them was observed (Figure 5). However, the association could not be found in cancer mortality (HR = 0.97, 95% CI = [0.88, 1.07], *I*^2^ = 44.8%, *p* = 0.041; Figure 6). Publication biases were observed in the cancer mortality (Egger’s test: coefficient = −1.224, *t* = −2.44, *p* = 0.033). However, no trimming was performed and the data were unchanged after using the trim-and-fill method. As a result, the impact of publication bias was considered minimal and the results were consistent.

## 4. Discussion

The idea behind artificial sweeteners was originally to replace the sugar so that using these products would reduce caloric intake, result in weight loss and reduce diabetes mellitus’ incidence [16]. However, existing research has shown that instead of reducing the risk of certain chronic diseases such as obesity, insulin resistance, or coronary artery disease, the use of artificial sweeteners even increases the likelihood of these diseases [47,48,49]. The results of our research demonstrated that artificial sweeteners’ intake seemed not to increase the risk of overall cancer incidence and mortality. However, in Europe, the consumption of artificial sweeteners could increase cancer incidence. This may be related to the fact that European countries are shifting towards a nutritional approach that adopts healthier eating behaviors. Over the past 30 years, dietary consumption patterns in European countries have changed significantly, with the average intake of sugar decreasing and people opting for its alternatives instead [50].

Our meta-analysis found a J-shaped association between artificial sweeteners intakes and all-cause mortality, which was also observed in some original studies and other similar meta-analysis [41,51]. Many previous studies have suggested that a reverse causation was existed [37,45,46]. The participants with the highest intake of artificial sweeteners were more likely to be obese, hypertensive and experience hypercholesterolemia, leading these people to switch to non-caloric sweeteners. Correspondingly, those people consuming artificial sweeteners in small quantities are more likely to have healthier lifestyles and dietary habits. However, almost all of the original studies adjusted for energy metabolism such as BMI and found that the association was attenuated but still significant, suggesting that the association cannot simply be explained by reverse causality. Interestingly, one study mentions that the result may reflect a cognitive process in which artificial sweeteners considered “healthy” allow an excessive consumption of other “unhealthy” foods [46]. Meanwhile, residual confounding could be an alternative explanation. In particular, we did not observe a clear association between artificial sweetener intake and cancer mortality. However, some of the original studies of our meta-analysis observed the association [40]. Although it did not provide a plausible explanation for the corresponding results, other studies have suggested that this may be related to sugar-sweetened foods that may lead to more severe clinical outcomes for patients with colon cancer [36].

Existing evidence indicated that the artificial sweeteners’ intake could indirectly cause a reduction in sugar-sweetened beverages or foods, thus leading to a decrease in the cancer incidence rate related to them [18]. Although most of the literature included in our study regarded artificial sweeteners and sugar-sweetened beverages as control variables mutually, the direct link between artificial sweeteners and cancer could be masked. Meanwhile, our study did not found the difference in the impact of artificial sweeteners on obesity-related cancer and other cancers. The results of the Melbourne Collaborative Cohort Study indicated that an association between artificial sweeteners and obesity-related cancers was not observed [27]. However, the association was found in a recent study [25]. As mentioned above, although the purpose of artificial sweeteners was to reduce obesity by substituting them for sugar, existing evidence suggests that artificial sweeteners could induce metabolic syndrome and the development of obesity by altering the host microbiome and reducing body satiety [16]. Therefore, one explanation for the existence of this association may be driven by overweight-related metabolic disturbances, although BMI and weight gain were adjusted throughout the study [25]. In essence, despite great interest in the potential of low-calorie sweeteners to prevent obesity and its complications, we found little evidence to support their health benefits. It also appears to have limited effects on blood glucose and lipids [52].

In subgroup analyses, we evaluated the effect of artificially sweetened beverages or aspartame intake alone. Aspartame, a well-known artificial sweetener, is now used as a sweetener and flavor enhancer in six thousand food products worldwide [33]. Notably, although aspartame is one of the most studied food additives, its safety remains controversial [31]. Formaldehyde, a metabolic byproduct of aspartame, is an established carcinogen that can cause DNA damage, chromosomal aberrations and mitotic errors [53,54]. Soffritti et al. found that it plays a major role in aspartame-induced carcinogenesis in the liver and lungs in mice [55]. Our meta-analysis also found a specific effect of aspartame intake on cancer. However, although aspartame exposure proved to increase incidence of lymphoma and leukemia in rats with a dose–response relationship in an Italian study, the European Food Safety Authority dismissed the findings due to the high rates of infection and inflammation in these animals, as well as the uncertainty of diagnosis [56,57]. In addition, recent studies have suggested that the measurements of ASBs might not be sufficient to accurately describe the overall dietary exposure to artificial sweeteners [25]. Through subgroup analysis, we found that although the effects of artificial sweeteners on cancer incidence were different based on the different intake type, a mixed intake of artificial sweeteners could increase the risk of cancer incidence, which seems to confirm the above view. Of course, given that there are fewer studies on aspartame or a mixed intake of artificial sweeteners, we hope to have more data in the future to clarify their relationship with cancer. 

Some limitations existed in our study. Although almost all of the original literature included are large-scale, prospective studies with long-term follow-up, the reliability of causal conclusions may not be as robust because they are all observational studies. Although possible confounding factors were adjusted in the original studies, the findings should be cautiously interpreted given the existence of residual confounding. Meanwhile, more data are also required to evaluate the effects of other artificial sweeteners on cancer.

## 5. Conclusions

Our meta-analysis indicated that artificial sweeteners’ intake could increase the risk of all-cause mortality, but the relationship was not observed in the risk of overall cancer incidence and mortality. However, in Europe, the consumption of artificial sweeteners could increase cancer incidence. More data from well-conducted studies and clinical trials are required to confirm the association.

## Figures and Tables

**Table 1:** Details of included studies.

Author	Year	Region	Sample Size	NOS	Cancer Type	Type of Artificial Sweetener Intake	Aspartame Intake Only	Follow-Up Time	Dose of Artificial Sweetener	Outcome
Heath A. K. et al [26].	2021	Europe	281,483	8	Kidney Cancer	ASBs alone	No	Incidence (15 years); Mortality (16 years)	per 100 g/day increment	Incidence/Cancer Mortality
Romanos-Nanclares A. et al [22].	2021	US	175,798	9	Breast Cancer	ASBs alone	No	36 years	≥1/day	Incidence
Debras C. et al [25].	2022	France	102,865	9	Cancer (unclassified)	Mixed intake	Yes	7.8 years	>17.44 mg/day in men and >19.00 mg/day in women	Incidence
Chazelas E. et al [24]. (1).	2019	France	101,257	9	Breast Cancer	ASBs alone	No	5.1 years	>7.9 mL/day in men and >11.6 mL/day in women	Incidence
Chazelas E. et al [24]. (2).					Colorectal Cancer					
Chazelas E. et al [24]. (3).					Prostate Cancer					
Navarrete-Munoz E. M. et al [32].	2016	Europe	477,199	9	Pancreatic Cancer	ASBs alone	No	11.60 years	>92.2 g/day	Incidence
Schernhammer E. S. et al [33].	2012	US	125,028	9	Hematopoietic Malignancy	Mixed intake	Yes	22 years	^†^	Incidence
Hodge A. M. et al [27]. (1).	2018	Australia	35,593	9	Prostate Cancer	ASBs alone	No	9–17 years	≥1/day	Incidence
Hodge A. M. et al [27]. (2).					Ovary Cancer					
Hodge A. M. et al [27]. (3).					Kidney Cancer					
Hodge A. M. et al [27]. (4).					Colorectal Cancer					
Hodge A. M. et al [27]. (5).					Breast Cancer					
Hodge A. M. et al [27]. (6).					Endometrium Cancer					
Hodge A. M. et al [27]. (7).					Gastric Cancer					
Hur J. et al [28].	2021	US	95,464	8	Colorectal Cancer	ASBs alone	No	24 years	≥2 servings/day	Incidence
Bassett J. K. et al [23].	2019	Australia	35,109	9	Cancers not related to obesity	ASBs alone	No	19 years	>1/day	Incidence
Stepien M. et al [34].	2016	Europe	477,206	7	Liver Cancer	ASBs alone	No	11.4 years	per 1 serving/day increment	Incidence
McCullough M. L. et al [31].	2014	US	100,442	9	Hematopoietic Malignancy	Mixed intake	Yes	10 years	≥1 can/day	Incidence
Lim U. et al [30]. (1).	2006	US	473,984	8	Hematopoietic Malignancy	Mixed intake	Yes	5 years	≥600 mg/d	Incidence
Lim U. et al [30]. (2).					Gliomas					
Inoue-Choi M. et al [29].	2013	US	23,039	8	Endometrial Cancer	ASBs alone	No	24 years	2.8–64.1 servings/week	Incidence
Zamora-Ros R. et al [42].	2022	Europe	450,064	8	Thyroid cancer	ASBs alone	No	14 years	43.0–3389.5 mL/d	Incidence
Guercio B. J. et al [36].	2018	US	1018	8	Colorectal Cancer	ASBs alone	No	10 months	≥2 servings/day	Cancer Mortality
Farvid M. S. et al [35].	2021	US	8863	8	Breast Cancer	ASBs alone	No	11.5 years	>3 servings/week	Cancer Mortality
Zhang Y. B. et al [39].	2020	US	31,402	8	Cancer (unclassified)	ASBs alone	No	7.9 years	≥2 servings/day	Cancer/All-Cause Mortality
Zoltick E. S. et al [40].	2021	US	1463	9	Colorectal Cancer	ASBs alone	No	8.0 years	per 1 serving/day increment	Cancer Mortality
Mullee A. et al [38]. (1).	2019	Europe	252,357	9	Colorectal Cancer	ASBs alone	No	16.4 years	≥1 servings/day	Cancer/All-Cause Mortality
Mullee A. et al [38]. (2).					Breast Cancer					
Mullee A. et al [38]. (3).					Prostate Cancer					
Malik V. S. et al [37]. (1).	2019	US	85,030	9	Lung Cancer	ASBs alone	No	34 years	≥2 servings/day	Cancer/All-Cause Mortality
Malik V. S. et al [37]. (2).					Colorectal Cancer					
Malik V. S. et al [37]. (3).					Breast Cancer					
Malik V. S. et al [37]. (4).					Prostate Cancer					
Liu D. et al [41].	2022	Europe	51,874	9	Cancer (unclassified)	ASBs alone	No	7.0 years	>4.5 servings /day	Cancer/All-Cause Mortality
Vyas A. et al [44].	2014	Columbia	59,614	9	-	ASBs alone	No	8.7 years	≥2/day	All-Cause Mortality
Anderson J. J. et al [45].	2020	Europe	198,285	9	-	ASBs alone	No	7 years	>2/day	All-Cause Mortality
Paganini-Hill A. et al [46].	2007	US	13,624	8	-	ASBs alone	No	23 years	>1 can/week	All-Cause Mortality
Mossavar-Rahmani Y. et al [43].	2019	US	81,714	8	-	ASBs alone	No	11.9 years	≥2/day	All-Cause Mortality

ASBs: artificially sweetened beverages; NOS: Newcastle–Ottawa scale. ^†^: Aspartame intake was divided into 5 categories by quantiles, but specific values for each category were not provided.

**Table 2:** Subgroup analyses were performed for cancer incidence and mortality.

Outcome	Sub-grouped by	No. of Studies	HR/RR	95% CI	Heterogeneity *I*^2^ (%)*, p*
Incidence	Region				
	Europe	6	1.07	(1.02, 1.12)	25.8%, 0.223
	Americas	6	0.97	(0.92, 1.02)	0.0%, 0.476
	Oceania	2	0.99	(0.85, 1.16)	25.8%, 0.223
	Cancer type				
	Obesity-related cancers	7	1.01	(0.94, 1.09)	51.7%, 0.011
	Cancers not related to obesity	8	1.04	(0.99, 1.09)	0.0%, 0.617
	Type of artificial sweetener intake				
	ASBs alone	10	1.02	(0.97, 1.08)	42.3%, 0.027
	Mixed intake	4	1.09	(1.01, 1.18)	0.0%, 0.513
	Aspartame intake only				
	Yes	4	1.10	(1.01, 1.19)	0.0%, 0.467
	No	10	1.02	(0.97, 1.08)	42.3%, 0.027
mortality	Region				
	Europe	3	1.05	(0.99, 1.13)	0.0%, 0.502
	Americas	5	0.91	(0.78, 1.06)	55.7%, 0.027
	Cancer type				
	Obesity-related cancers	5	0.97	(0.84, 1.13)	46.7%, 0.059
	Cancers not related to obesity	2	1.01	(0.89, 1.15)	54.4%, 0.139

ASBs: artificially sweetened beverages; HR: hazard ratio; RR: relative risk; CI: Confidence Interval.

**Table 3:** Meta-regression for cancer incidence.

Variables	*I*^2^ (%)	Adj R^2^	Exp (b)	Std. Err.	*t*	*p*	95% CI
Region (Europe)	16.22	35.98	1.11	0.06	2.09	0.048	(1.01, 1.23)
Region (Oceania)			1.04	0.08	0.55	0.588	(0.89, 1.22)
Cancer type	35.32	−46.15	0.96	0.05	−0.72	0.476	(0.86, 1.08)
Type of artificial sweetener intake	34.14	14.61	0.95	0.05	−0.93	0.360	(0.84, 1.07)
Aspartame intake only	34.82	16.88	1.06	0.06	0.97	0.341	(0.94, 1.20)

CI: Confidence Interval.

## Supplementary Materials

The following supporting information can be downloaded at: https://www.mdpi.com/article/10.3390/nu14183742/s1, Table S1: Search strategy (PubMed).
