# Supplementary material for: Can Artificial Sweeteners Increase the Risk of Cancer Incidence and Mortality: Evidence from Prospective Studies
Source: Nutrients. 2022 Sep 10;14(18):3742. doi: 10.3390/nu14183742 (PMC9506029; doi:10.3390/nu14183742)
Supplement: Supplementary file 1 [file nutrients-14-03742-s001.zip › nutrients-1872853-supplementary.pdf]

Table S1 Search strategy (PubMed)

Exposure	("sweetening agents"[Pharmacological Action] OR "sweetening agents"[MeSH Terms] OR ("sweetening"[All Fields] AND "agents"[All Fields]) OR "sweetening agents"[All Fields] OR ("artificial"[All Fields] AND "sweetener"[All Fields]) OR "artificial sweetener"[All Fields] OR ("non nutritive sweeteners"[MeSH Terms] OR ("non nutritive"[All Fields] AND "sweeteners"[All Fields]) OR "non nutritive sweeteners"[All Fields] OR ("non"[All Fields] AND "nutritive"[All Fields] AND "sweeteners"[All Fields]) OR "non nutritive sweeteners"[All Fields]) OR ("aspartame"[MeSH Terms] OR "aspartame"[All Fields] OR "aspartam"[All Fields] OR "aspartame s"[All Fields]) OR ("saccharin"[MeSH Terms] OR "saccharin"[All Fields] OR "saccharine"[All Fields] OR "saccharins"[All Fields]) OR ("cyclamates"[MeSH Terms] OR "cyclamates"[All Fields] OR "cyclamate"[All Fields] OR "cyclamic"[All Fields]) OR ("stevia"[MeSH Terms] OR "stevia"[All Fields] OR "stevias"[All Fields]) OR ("trichlorosucrose"[Supplementary Concept] OR "trichlorosucrose"[All Fields] OR "sucralose"[All Fields]) OR ("acesulfame"[All Fields] OR "acesulphame"[All Fields]) OR ("artificially sweetened beverages"[MeSH Terms] OR ("artificially"[All Fields] AND "sweetened"[All Fields] AND "beverages"[All Fields]) OR "artificially sweetened beverages"[All Fields]) OR ("artificially sweetened beverages"[MeSH Terms] OR ("artificially"[All Fields] AND "sweetened"[All Fields] AND "beverages"[All Fields]) OR "artificially sweetened beverages"[All Fields] OR ("artificially"[All Fields] AND "sweetened"[All Fields] AND "beverage"[All Fields]) OR "artificially sweetened beverage"[All Fields]))
Outcome	("cancer s"[All Fields] OR "cancerated"[All Fields] OR "canceration"[All Fields] OR "cancerization"[All Fields] OR "cancerized"[All Fields] OR "cancerous"[All Fields] OR "neoplasms"[MeSH Terms] OR "neoplasms"[All Fields] OR "cancer"[All Fields] OR "cancers"[All Fields] OR ("cysts"[MeSH Terms] OR "cysts"[All Fields] OR "cyst"[All Fields] OR "neurofibroma"[MeSH Terms] OR "neurofibroma"[All Fields] OR "neurofibromas"[All Fields] OR "tumor s"[All Fields] OR "tumoral"[All Fields] OR "tumorous"[All Fields] OR "tumour"[All Fields] OR "neoplasms"[MeSH Terms] OR "neoplasms"[All Fields] OR "tumor"[All Fields] OR "tumour s"[All Fields] OR "tumoural"[All Fields] OR "tumourous"[All Fields] OR "tumours"[All Fields] OR "tumors"[All Fields]) OR ("carcinoma"[MeSH Terms] OR "carcinoma"[All Fields] OR "carcinomas"[All Fields] OR "carcinoma s"[All Fields]) OR ("neoplasm s"[All Fields] OR "neoplasms"[MeSH Terms] OR "neoplasms"[All Fields] OR "neoplasm"[All Fields]))
